# Profit distribution mechanism of agricultural supply chain based on fair entropy

**DOI:** 10.1371/journal.pone.0271693

**Published:** 2022-07-25

**Authors:** Fuzhen Gu, Xun Yu

**Affiliations:** 1 School of Economics and Management, Panzhihua University, Panzhihua, Sichuan Province, China; 2 School of Economics and Management, Heilongjiang Institute of Technology, Harbin, Heilongjiang Province, China; Al Mansour University College-Baghdad-Iraq, IRAQ

## Abstract

This paper constructs a profit distribution model under centralized decision-making by taking the secondary agricultural supply chain as an example. Moreover, it introduces resources and risk as two factors to form significant weights and then designs a fair profit distribution mechanism of the supply chain according to the weights. Finally, this paper solves the optimal solution of the profit distribution coefficient by using fair entropy. Research results find that the distribution according to the optimal profit distribution coefficient can maximize the profit of each actor and achieves the matching of returns and inputs. Therefore, this paper puts forward some suggestions that include establishing a precise distribution mechanism based on contracts or property rights definitions, considering the cost and importance weight of each actor’s input when formulating the profit distribution coefficient, and paying attention to information sharing.

## 1. Introduction

The supply chain is a long-term and continuous contractual relationship established by enterprises with independent decision-making power to achieve strategic goals. The agricultural product supply chain is a comprehensive application of supply chain thinking in the production and circulation of agricultural products [1]. However, compared with the supply chain of industrial products, the agricultural product supply chain is characterized by many participants, a low degree of production organization, and relatively scattered consumption terminals. These characteristics lead to large differences in the significant and status of farmers as producers, distributors and retailers, and there is no incentive mechanism to promote information exchange among the participating subjects, which dramatically increases the difficulty of coordinating of the agricultural product supply chain. Therefore, when the random changes in consumer demand for agricultural products lead to drastic price fluctuations, the whole agricultural product supply chain is prone to a crisis of breakage. Changes in policies and information technology are likely to restructure the agricultural supply chain.

Since all partners’ primary goal in the supply chain is maximizing their own interests, and each member enterprise is an independent economic entity, no partner is willing to sacrifice their interests to enhance the interests of other partners. Therefore, the supply chain management of agricultural products cannot merely refer to the methods and concepts of industrial supply chain management. It is necessary to form the corresponding operation and cooperation method according to the characteristics of the agricultural supply chain in order to realize integration [2] (Zhang, 2011). The method can also combine the production, circulation and marketing of agricultural products with contracts, and establish a long-term strategic cooperation relationship. In addition, the distribution of benefits is the focal issue of reducing the opportunistic behavior of participants and achieving efficient allocation of resources [3]. A fair and reasonable benefit distribution mechanism is conducive to enhancing the agility of the entire agricultural supply chain, reducing internal transaction costs, promoting the stability and closeness of cooperative relationships, and thus generating collaborative benefits.

Given the above, this paper takes the secondary agricultural product supply chain as an example, constructs a profit distribution model under centralized decision-making, introduces the dual factors of resources and risks to form importance weights, and designs a fair profit distribution mechanism of the supply chain according to the weights. Finally, we solve the optimal solution of profit distribution coefficients using fair entropy and verify the model with an example. It is expected to form an effective profit distribution mechanism that can generate intrinsic incentives. The decision-making of each participant can increase their own profits while promoting the matching of agricultural product supply and market demand to guarantee the stable and orderly development of agriculture.

This paper is organized sequentially (a) to analyze the main features of the agricultural product supply chain, (b) to develop a profit distribution model for the agricultural product supply chain, (c) to solve the profit distribution model to obtain the optimal profit distribution coefficient, (d) to conduct a case study to verify and illustrate the profit distribution model, and (e) to sum up conclusions and provide suggestions.

## 2. Main features of the agricultural products supply chain and analysis of the relationship between nodal units

### (1) Main features of agricultural products supply chain

Agricultural products are relatively dependent on natural forces, the natural environment, climate, energy use and crop vitality in their production process [4–7]. The production of agricultural products has the characteristics of seasonality, regionality and dispersion. In addition, the freshness of agricultural products must be maintained. Agricultural products are relatively dependent on natural forces, the natural environment and crop vitality in their production process. Agricultural product supply chain can be simply described as the flow of products from the earliest process of production to final customers [8]. So far, there is no specific and unified definition of the agricultural product supply chain. This paper conceptualizes the agricultural products supply chain as an interdependent and consensus vertical network organization system including suppliers of agricultural production materials, planting and breeding producers, processing enterprises, distributors and consumers, according to the relevant descriptions of domestic and foreign scholars. It covers all relationships in the exchange process, in which the government’s domination plays a vital role and has national security characteristics.

#### a. Extremely complex relationship

Individual businesses gradually realize that they no longer compete as stand-alone entities, but as a participant in supply chains [9]. The production of agricultural products requires the input of production materials and industrial products to improve the production efficiency of agricultural products, industrial processing to realize the value-added of agricultural products, and finally create the value of agricultural products through the circulation links. Therefore, the agricultural product supply chain has many links, in which numerous participants are involved whose strength and status are exceedingly asymmetrical. The interaction between these participating subjects leads to a complex relationship in the agricultural products supply chain.

#### b. Loose and unstable relationship between enterprises at each node

Due to highly scattered consumers, small production and large market, there is information asymmetry between nodes in the agricultural supply chain [10, 11]. For instance, farmers without sufficient knowledge of the market may sell the produce at a meagre price which actually deserves a high price [12] (Gardas et al., 2019). Moreover, it is difficult for each node enterprise to fully grasp the market supply and demand information and industry information. The cooperative relationship between node enterprises is relatively loose, and the overall connection of the supply chain has randomness. In addition, many changes in the external environment that lead to the reorganization of supply chain relationships, such as policies, consumer demand, changes in information and logistics technology, the involvement of external investors, natural disasters and so on.

#### c. Higher asset specificity

Agricultural products are characterized by solid regionalism, small production scale and diverse consumer demands. It requires specialization in logistics storage, transportation, distribution, information technology and logistics information management. Perishability is another feature that should be considered in agricultural supply chain, and keeping products fresh can bring additional profits [13]. For some certain products, it’s necessary to ensure the quality and freshness of the products through cold chain technology, so that the agricultural products can be circulated and traded among different regions. Besides, agricultural products have a relatively long production cycle and a long payback period, and are also limited by natural conditions such as seasons and climates. These characteristics lead to excellent resistance for companies to enter and leave the industry.

### (2) Research on the exchange relationship between node enterprises in the supply chain of agricultural products

The theory of transaction cost economics is established from the three transaction dimensions concluding asset features, opportunistic behavior assumptions and transaction uncertainty. The theory study the commodity exchange relationship between node enterprises in the agricultural product supply chain. The theory also explore what kind of transaction mode and organization form can prevent opportunistic behavior in order to reduce transaction costs. However, the theory cannot explain the functions and roles of the information and emotion exchange, trust, identification, sharing, dependence, cooperative innovation and other elements among the various subjects in the process of supply chain development [14]. In the article "Economic Action and Social Structure", the sociologist Granovetter proposed the theory of "social embeddedness", arguing that in economics, "economical people" are "lowly socialized people". The economic actors are "embedded" in the actual social structure and social relationship network, and their behavioral choices can be based on their subjective purposes. Therefore, economic transaction relations and social interaction relations exist simultaneously among the node enterprises of the agricultural product supply chain [15]. Through the mutual combination of exclusive resources and specific economic transaction behaviors, the parties to the transaction obtain relationship rents that cannot be generated when they operate independently as single economic entities.

In the initial stage of building an agricultural product supply chain, the node enterprises mainly related to each other by contract, which led to their lack of mutual communication and understanding [16]. With repeated and continuous transactions, information and knowledge frequently spread between node enterprises. The degree of mutual awareness grows and relationship norms are gradually formed. All previous transaction experiences, social relationship backgrounds and expectations for future transactions between transaction subjects are embedded in this information and knowledge, and can provide a reference for each transaction [17]. Sharing real-time information aims to generate the synergy to improve economic benefits [18]. The dissemination of information and knowledge among supply chain subjects deepens the emotion and understanding of each other and establishes their trust relationships. Under the domination of such trust, sharing knowledge, information and resources, and collaborative innovation among supply chain subjects will gain higher incentives. Node enterprises will develop better self-discipline and gradually form an operating mechanism of risk sharing, complementary advantages, and resource sharing. Consequently, the entire agricultural supply chain becomes more efficient and generate synergistic benefits [19]. Therefore, the relationship norm is the common expectation of all parties in the node-enterprise relationship for appropriate behavior. The distinctive feature is the sharing of behavioral norms and values. It is an endogenous mechanism, conducive to improving the transaction performance of the agricultural supply chain.

The relational exchange theory suggests that the variables of relational elements in commodity transaction relations determine the identification of various exchange relations. In the process from individual transaction to vertical integration, the closer to the individual transaction, the more market transaction characteristics are presented in the exchange relationship. The contract transaction characteristics are presented in short-term commodity exchange relations with lower relational elements. The closer to the vertical integration, the more normative characteristics of the relationship will be presented. Social interaction will gradually increase. Besides, the closeness, stability and level of cooperation between node enterprises will gradually strengthen with better economic performance and lower transaction costs.

## 3. Profit distribution model of agricultural product supply chain

From the overall point of view, the interest of the agricultural product supply chain can be divided into three parts: the subject of interest, the object, and the intermediary.

As the subject of interest, farmers are the foundation and premise of the agricultural product chain, the provider of agricultural products, and the starting point and core part of the chain. As the core of the supply chain, it gathers the resources of the entire chain. Its operation affects the continuity of the chain and the profitability of each link in the chain. Stakeholders should manage the development of the entire chain with a suitable operating mechanism and a scientific operating plan, which can promote the interests of each enterprise in the chain.

Participating enterprises in each link of the agricultural production chain are the object of interest and the objects of interest distribution. The supply chain of agricultural products must rely on these objects to achieve the ultimate goal. They jointly provide direct and indirect benefits to the entire chain. Before joining the agricultural product supply chain, the interests of an enterprise are mainly the profits obtained from its business sales minus costs. However, after joining the supply chain, various interest objects collectively generate the interests of the enterprise.

An interest intermediary is a part of the intermediate links in the supply chain, including middlemen and logistics. Since there are many exchanges and communications in the operation of the agricultural product supply chain, we need to rely on the management system to maintain the sustainable development of the chain. In other words, the role of interest intermediaries is to provide norms and management to ensure the development of the chain and improve adaptability in the changing environment.

In order to ensure the balanced distribution of the interests of each enterprise entity in the chain, a reasonable interest distribution mechanism can be formulated.

### (1) Model assumptions and variable descriptions

Research is carried out on the secondary agricultural product supply chain composed of a specialized farmers’ cooperative and a single wholesaler. At the beginning of the production season, the specialized farmers’ cooperative organizes its members to purchase agricultural materials and decides the production type and output. After the production season, wholesalers purchase the agricultural products produced by the cooperative in bulk and then decide the selling price for market sales. In order to facilitate quantitative analysis, the following assumptions are made:

**Hypothesis 1**. Both the cooperative and the wholesaler are risk-neutral and rational decision-makers and share all information, regardless of the impact of emotional factors on both parties’ business.
**Hypothesis 2.** The loss of agricultural products during transportation and the impact of out-of-stocks on the cooperative and the wholesaler are not considered. Besides, the residual value of unsold agricultural products is zero.
**Hypothesis 3.** The market demand function satisfies *Q*(*P*) = *m* − *λP*, where *m* represents the market size, *λ* represents the price elasticity of demand, and the value is determined by the nature of the market.

The subscript “1” represents specialized farmers’ cooperatives, and the subscript “2” represents wholesalers. The production cost per unit of agricultural products of cooperatives is *C*_1_. The wholesale price per unit of agricultural products of cooperatives is *P*_1_. Similarly, the sales cost per unit of agricultural products of wholesalers is *C*_2_, including inventory, transportation, and other costs, while the selling price per unit of agricultural products of wholesalers is *P*_2_. *R*_1_ and *R*_2_ represents the profit of the cooperative and the wholesaler, respectively.

### (2) Profit distribution model of decentralized agricultural product supply chain

In the decentralized agricultural product supply chain, there is only a market transaction relationship between the cooperative and the wholesaler, that is, both sides only agree on the transaction price of agricultural products and there is no other constraint mechanism. Moreover, both parties pursue the maximization of personal benefits. The cooperative decides the wholesale price per unit of agricultural products, and then the wholesaler decides the sales price and order quantity. The profit function of the wholesaler is

$$R_{2}(P_{2})=(P_{2}-P_{1}-C_{2})*(m-\lambda P_{2})$$ (1)

When the expected profit of the wholesaler is maximized, it satisfies that $\frac{\partial R_{2}}{\partial P_{2}}=0$. Hence, the optimal sales price determined is

$${\bar{P}}_{2}=\frac{m+\lambda (P_{1}+C_{2})}{2\lambda }$$ (2)

Substituting (2), under decentralized decision-making, the profit function of the cooperative is

$$\begin{array}{l} R_{1}(P_{1})=(P_{1}-C_{1})*(m-\lambda P_{2})=(P_{1}-C_{1})*(m-\lambda \frac{m+\lambda (P_{1}+C_{2})}{2\lambda })\\ =(P_{1}-C_{1})\frac{m-\lambda (P_{1}+C_{2})}{2} \end{array}$$ (3)

Similarly, when the expected profit of the cooperative is maximized, it satisfies that $\frac{\partial R_{1}}{\partial P_{1}}=0$. Therefore, the optimal wholesale price is

$${\bar{P}}_{1}=\frac{m+\lambda (C_{1}-C_{2})}{2\lambda }$$ (4)

Substituting Eqs (2) and (4) into Eqs (1) and (3), the maximum expected profit of the cooperative and the wholesaler is obtained as

$$\left\{\begin{array}{c} {\bar{R}}_{1}=\frac{{\left[m-\lambda (C_{1}+C_{2})\right]}^{2}}{8\lambda }\\ {\bar{R}}_{2}=\frac{{\left[m-\lambda (C_{1}+C_{2})\right]}^{2}}{16\lambda } \end{array}\right.$$ (5)

The maximum expected profit of the decentralized agricultural product supply chain system is

$$\bar{R}={\bar{R}}_{1}+{\bar{R}}_{2}=\frac{3{\left[m-\lambda (C_{2}+C_{1})\right]}^{2}}{16\lambda }$$ (6)

### (3) Profit distribution model of integrated agricultural product supply chain

The integrated agricultural product supply chain is formed by establishing a specific restraint mechanism between cooperatives and wholesalers. This paper sets the restraint mechanism as a revenue-sharing contract. As decision-making bodies with integrated interests, cooperatives and wholesalers jointly set the sales price of agricultural products and then share the excess profits generated by the formation of the integrated agricultural product supply chain according to a certain proportion. Therefore, the profits of cooperatives and wholesalers are composed of two parts. The first part is the maximum profit obtained in the decentralized agricultural supply chain, and the second is the excess profit distributed according to the coefficient. *φ* is the excess profit distribution coefficient. P′ represents the market sales price under the centralized decision-making of cooperatives and wholesalers.

Under centralized decision-making, the profit function of the agricultural product supply chain system is

$$R^{\prime }=(P^{\prime }-C_{1}-C_{2})*(m-\lambda P^{\prime })$$ (7)

When the expected profit of the integrated agricultural product supply chain is maximized, it satisfies that $\frac{\partial R^{\prime }}{\partial P^{\prime }}=0$. The optimal sales price determined is

$$\bar{P^{\prime }}=\frac{m+\lambda (C_{1}+C_{2})}{2\lambda }$$ (8)

Substituting Eq (8) into Eq (7), the maximum expected profit of the system is obtained as

$$\bar{R^{\prime }}=\frac{{\left[m\text{-}\lambda (C_{1}+C_{2})\right]}^{2}}{4\lambda }$$ (9)

The excess profits formed by the establishment of the integrated agricultural product supply chain and the excess profits shared by the cooperatives and wholesalers are

$$\left\{\begin{array}{c} \Delta \text{R}=\frac{{\left[m\text{-}\lambda (C_{1}+C_{2})\right]}^{2}}{16\lambda }\\ \Delta {\text{R}}_{\text{1}}=\varphi *\frac{{\left[m\text{-}\lambda (C_{1}+C_{2})\right]}^{2}}{16\lambda }\\ \Delta {\text{R}}_{\text{2}}=(1-\varphi )*\frac{{\left[m\text{-}\lambda (C_{1}+C_{2})\right]}^{2}}{16\lambda } \end{array}\right.$$ (10)

According to the revenue sharing contract, the maximum profit of the cooperative under centralized decision-making is

$$\bar{{R^{\prime }}_{1}}={\bar{R}}_{1}+\Delta R_{1}=\frac{(\varphi +2){\left[m\text{-}\lambda (C_{1}+C_{2})\right]}^{2}}{16\lambda }$$ (11)

The maximum profit of the wholesaler under centralized decision-making is

$$\bar{{R^{\prime }}_{2}}={\bar{R}}_{2}+\Delta R_{2}=\frac{(2-\varphi ){\left[m\text{-}\lambda (C_{1}+C_{2})\right]}^{2}}{16\lambda }$$ (12)

## 4. Construction and solution of profit distribution coefficient model considering fairness

To maintain the stable existence of the integrated agricultural product supply chain, the profit of each participant should increase compared with the decentralized decision-making, and the principle of fairness should be satisfied to the greatest extent. The fairness of profit distribution is reflected in matching members’ input and income returns so that their psychological expectations are satisfied [20]. In cooperative game theory, the traditional Shapley method only considers the contribution of each member when distributing profits, and may not be suitable for the "farmer-cooperative-supermarket" model [21]. By modifying the classical Shapley value, stakeholders achieved their profit share based on their marginal contribution, risk potential, and value-added contribution [22]. Referring to the practice of literature [23], since the profits distributed by cooperatives and wholesalers are excess profits generated after forming the integrated supply chain, fairness is reflected in that the excess profit rates of both parties are equal. The excess profit rate is defined as

$$e_{i}=\frac{\Delta R_{i}}{\alpha_{i}C_{i}}(i=1,2)$$ (13)

where Δ*R*_*i*_ is the excess profit shared by the member, *α*_*i*_ is the member’s weight in the integrated supply chain, and *C*_*i*_ is the member’s cost. When designing the importance weight, the reference only considers the resource input of each member in the supply chain, and the analytical solution of the model is not obtained. However, an essential principle of profit distribution is matching risks and benefits [23]. Members with more significant risks should be compensated for more benefits to enhance their enthusiasm for participating in cooperation. Therefore, this paper introduces the risk factor on its basis, calculates the importance weight of cooperatives and wholesalers through the resource factor and risk factor, and obtains the optimal solution of the profit distribution coefficient.

### (1) Weight calculation of resource factor

The key to the integrated agricultural product supply chain is that each participant invests valuable resources, so the resource factor is the primary consideration for the importance weight. The expert scoring method is used to determine the weights of resource factors for cooperatives and wholesalers in the integrated supply chain. Assuming that *m* experts evaluate the contribution of the input resources of cooperatives and wholesalers to the establishment of an integrated supply chain. The following evaluation matrix can be obtained:

$$\Omega_{1}=\left[\begin{array}{cc} \begin{array}{c} W_{11}\\ W_{21}\\ \vdots \\ W_{m1} \end{array} & \begin{array}{c} W_{12}\\ W_{22}\\ \vdots \\ W_{m2} \end{array} \end{array}\right]$$ (14)

where *W*_*i*1_ indicates the rating on the resources of cooperatives of expert *i*, *W*_*i*2_ indicates the rating on the resources of wholesalers for *W*_*i*1_, *W*_*i*2_ = {1 2 3 … 8 9}. The larger the number, the greater the importance, i.e., 1 means not important, 9 means extremely important [24].

Then we average expert ratings for cooperatives and wholesalers. *W*_1_ is for the cooperative while *W*_2_ is for the wholesaler.


$$\left\{\begin{array}{c} W_{1}=\frac{1}{m}\sum_{i=1}^{m}W_{i1}\\ W_{2}=\frac{1}{m}\sum_{i=1}^{m}W_{i2} \end{array}\right.$$ (15)

Based on the average scores of the expert, the weights of resource factors for cooperatives and wholesalers are obtained. *d*_1_ is for the cooperative while *d*_2_ is for the wholesaler.


$$\left\{\begin{array}{c} d_{1}=\frac{W_{1}}{W_{1}+W_{2}}\\ d_{2}=\frac{W_{2}}{W_{1}+W_{2}} \end{array}\right.$$ (16)

### (2) Weight calculation of risk factor

Due to the characteristics of agricultural products and the specificity of some assets, each member of the integrated agricultural product supply chain has different risks. When calculating the importance weight, if the risk factor is not considered, it will make the members with high risk have a severe psychological imbalance, lose motivation and even seek compensation from other channels, thereby destroying the stability of the collaboration. Through the review and analysis of previous literature, a comprehensive conceptual risk classification framework for supply chain is proposed. The framework consists of the following dimensions: product characteristics, supply chain management process, supply chain infrastructure, external environment, and human resources [25]. As for the supply chain of agricultural products, The study constructs interrelationships among various risk factors, and concludes that extreme weather conditions and political and economic instability are the key threat [26]. Therefore, we use a fuzzy comprehensive evaluation method to determine the weights of risk factors for cooperatives and wholesalers. The five main risks affecting the agricultural product supply chain are agricultural product supply risk, market demand change risk, information technology risk, cooperation risk, and logistics and transportation risk. Among them, the supply risk comes from the seasonality of agricultural production. The fact that weather easily affects the output makes it difficult for agricultural product producers to guarantee the quantity and quality of products supplied. It is hard for agricultural product sellers to accurately predict the demand for a particular product because of the risk of changes in market demand caused by the uncertainty of consumer preferences. Information technology risk comes from two aspects. One is that the utilization rate of modern communication technology in agriculture is not high. The other is that sellers have no incentive to share information with producers. Cooperation risk means that unethical behaviors such as breach of contract and deception may occur between suppliers and sellers in the process of cooperation. Due to the characteristics of agricultural products that are perishable and easy to wear, there are potential logistics and transportation risks without an efficient logistics operation system.

Suppose the set X = {X_1_, X_2_, X_3_, X_4_, X_5_} = {agricultural product supply risk, market demand change risk, information technology risk, cooperation risk, logistics and transportation risk} represents the set of alternative objects, the set A = {*a*_1_, *a*_2_, *a*_3_, *a*_4_, *a*_5_} represents the weight vector set of risks, and the set D represents the level set of risks, where D = {none, low, relatively low, medium, relatively high, high} = {0, 0.1, 0.3, 0.5, 0.7, 0.9}. According to the experts scores, the fuzzy evaluation matrix is obtained as follows.

$$\text{P}=\left[\begin{array}{cccc} p_{11} & p_{12} & \cdots & p_{15}\\ p_{21} & p_{22} & \cdots & p_{25}\\ \vdots & \vdots & \ddots & \vdots \\ p_{51} & p_{52} & \cdots & p_{55} \end{array}\right]$$ (17)

With the appropriate fuzzy synthesis operator for operation, the comprehensive evaluation matrix of risk is obtained as follows.


$$\begin{array}{l} \text{B}=A\cdot \text{P}=\left[\begin{array}{ccccc} a_{1} & a_{2} & a_{3} & a_{4} & a_{5} \end{array}\right]\left[\begin{array}{cccc} p_{11} & p_{12} & \cdots & p_{15}\\ p_{21} & p_{22} & \cdots & p_{25}\\ \vdots & \vdots & \ddots & \vdots \\ p_{51} & p_{52} & \cdots & p_{55} \end{array}\right]=\left[\begin{array}{ccccc} b_{1} & b_{2} & b_{3} & b_{4} & b_{5} \end{array}\right] \end{array}$$ (18)

The fuzzy evaluation matrix B is normalized and represented by B′, and the calculated risk coefficient of the integrated agricultural product supply chain is

$$\text{U}=\text{B'}\cdot {\text{D}}^{\text{T}}$$ (19)

*u*_1_ is the risk factor of cooperatives while *u*_2_ is the risk factor of wholesalers.


$$\left\{\begin{array}{c} u_{1}=\frac{U_{1}}{U_{1}+U_{2}}\\ u_{2}=\frac{U_{2}}{U_{1}+U_{2}} \end{array}\right.$$ (20)

### (3) Solving the optimal profit distribution coefficient based on fair entropy

Fairness entropy is an evolution of the concept of entropy in thermodynamics. It is a value that connects each member’s maximum profit and contribution to the integrated agricultural supply chain. The larger the fairness entropy value, the higher the fairness of profit distribution. The fairness entropy is introduced to measure the fairness of the profit distribution of cooperatives and wholesalers. The calculation formula of fairness entropy is defined as follows [22]:

$$H=-\frac{1}{Ln2}(\gamma_{1}Ln\gamma_{1}+\gamma_{2}Ln\gamma_{2})$$ (21)

*γ* represents the normalized result of the excess profit rate *e*, i.e., $\gamma_{i}=\frac{e_{i}}{e_{1}+e_{2}}(i=1,2)$. Therefore, we need calculate the excess profit rate of cooperatives and wholesalers.

Since the resource factor and risk factor are used to determine the importance weight jointly, we assume that the proportion of resource factor in the importance weight is *ρ*_1_ and the proportion of risk factor is set as *ρ*_2_ which satisfies *ρ*_1_ + *ρ*_2_ = 1. The importance weights of cooperatives and wholesalers in the integrated supply chain can be obtained:

$$\left\{\begin{array}{c} \alpha_{1}=\rho_{1}d_{1}+\rho_{2}u_{1}\\ \alpha_{2}=\rho_{1}d_{2}+\rho_{2}u_{2} \end{array}\right.$$ (22)

Substituting Eqs (10) and (22) into Eqs (13), we get the normalized excess profit rate for the cooperative and wholesaler.


$$\left\{\begin{array}{c} \gamma_{1}=\frac{\varphi \alpha_{2}C_{2}}{\varphi \alpha_{2}C_{2}+(1-\varphi )\alpha_{1}C_{1}}\\ \gamma_{2}=\frac{(1\text{-}\varphi )\alpha_{1}C_{1}}{\varphi \alpha_{2}C_{2}+(1-\varphi )\alpha_{1}C_{1}} \end{array}\right.$$ (23)

Substituting Eqs (23) into Eqs (21), when the value of the fair entropy is maximized, it satisfies that $\frac{\partial \text{H}}{\partial \varphi }=0$. It can be obtained by calculation

$$\begin{array}{l} \frac{\partial \text{H}}{\partial \varphi }=\text{-}\frac{1}{Ln2}\frac{\alpha_{2}C_{2}\alpha_{1}C_{1}}{{\left[\varphi \alpha_{2}C_{2}+(1-\varphi )\alpha_{1}C_{1}\right]}^{2}}*Ln\frac{\varphi \alpha_{2}C_{2}}{(1-\varphi )\alpha_{1}C_{1}}=0 \end{array}$$ (24)

Since *α*_2_, *C*_2_, *α*_1_, *C*_1_ are not zero, *α*_2_*C*_2_*α*_1_*C*_1_ ≠ 0. To make Eq (24) hold, it can only satisfy $\frac{\varphi \alpha_{2}C_{2}}{(1-\varphi )\alpha_{1}C_{1}}=1$. Hence, the optimal solution of the profit distribution coefficient can be obtained:

$$\varphi =\frac{\alpha_{1}C_{1}}{\alpha_{2}C_{2}+\alpha_{1}C_{1}}=\frac{1}{\frac{\alpha_{2}}{\alpha_{1}}*\frac{C_{2}}{C_{1}}+1}=\frac{1}{\frac{\alpha_{2}}{\alpha_{1}}/\frac{C_{1}}{C_{2}}+1}$$ (25)

Under the optimal profit distribution coefficient, the maximum profit that cooperatives and wholesalers can obtain in the integrated agricultural product supply chain is:

$$\left\{\begin{array}{c} \bar{{R^{\prime }}_{1}}={\bar{R}}_{1}+\Delta R_{1}\frac{(\frac{\alpha_{1}C_{1}}{\alpha_{2}C_{2}+\alpha_{1}C_{1}}+2){\left[m\text{-}\lambda (C_{1}+C_{2})\right]}^{2}}{16\lambda }\\ \bar{{R^{\prime }}_{2}}={\bar{R}}_{2}+\Delta R_{2}=\frac{(2-\frac{\alpha_{1}C_{1}}{\alpha_{2}C_{2}+\alpha_{1}C_{1}}){\left[m\text{-}\lambda (C_{1}+C_{2})\right]}^{2}}{16\lambda } \end{array}\right.$$ (26)

## 5. Result analysis and calculation verification

The following results and propositions are obtained by analyzing the optimal solution of the profit distribution coefficient obtained by formula (25). As shown in Table 1.

**Table 1:** The analysis of profit distribution coefficient result.

The relationship between $\frac{\alpha_{2}}{\alpha_{1}}$ and $\frac{C_{1}}{C_{2}}$	$\varphi =\frac{1}{\frac{\alpha_{2}}{\alpha_{1}}/\frac{C_{1}}{C_{2}}+1}$
$\frac{\alpha_{2}}{\alpha_{1}}/\frac{C_{1}}{C_{2}}\to \infty$	*φ* → 0
$\frac{\alpha_{2}}{\alpha_{1}}>\frac{C_{1}}{C_{2}}$	$\varphi <\frac{1}{2}$
$\frac{\alpha_{2}}{\alpha_{1}}=\frac{C_{1}}{C_{2}}$	$\varphi =\frac{1}{2}$
$\frac{\alpha_{2}}{\alpha_{1}}<\frac{C_{1}}{C_{2}}$	$\varphi >\frac{1}{2}$
$\frac{\alpha_{2}}{\alpha_{1}}/\frac{C_{1}}{C_{2}}\to 0$	*φ* → 1

**Proposition 1.** The ratio of the profit distribution coefficient to the importance weight changes in the opposite direction, and the rate of change decreases.
**Proof.** Assume that the ratio of input costs remains the same, i.e., $\frac{C_{2}}{C_{1}}=k$. It can be found that, as $\frac{\alpha_{2}}{\alpha_{1}}$ approaches zero, the profit distribution coefficient *φ* approaches one. Under this condition, the contribution of cooperatives to the integrated agricultural product supply chain is much higher than that of wholesalers, and it is entirely irreplaceable. Therefore, cooperatives have absolute control over the integrated supply chain’s operation, coordination, and profitability, and almost all of the excess profits will be occupied by cooperatives.

On the contrary, as $\frac{\alpha_{2}}{\alpha_{1}}$ approaches infinity, the profit distribution coefficient *φ* approaches zero. The contribution of cooperatives to the integrated agricultural product supply chain is quite low under this condition. There is little impact on the cooperative relationship and supply chain operation. However, the wholesalers will dominate the transaction and obtain almost all excess profits with channel and information resources. In contrast, cooperatives can only obtain profits equivalent to those under the decentralized agricultural supply chain.

**Fig 1:**
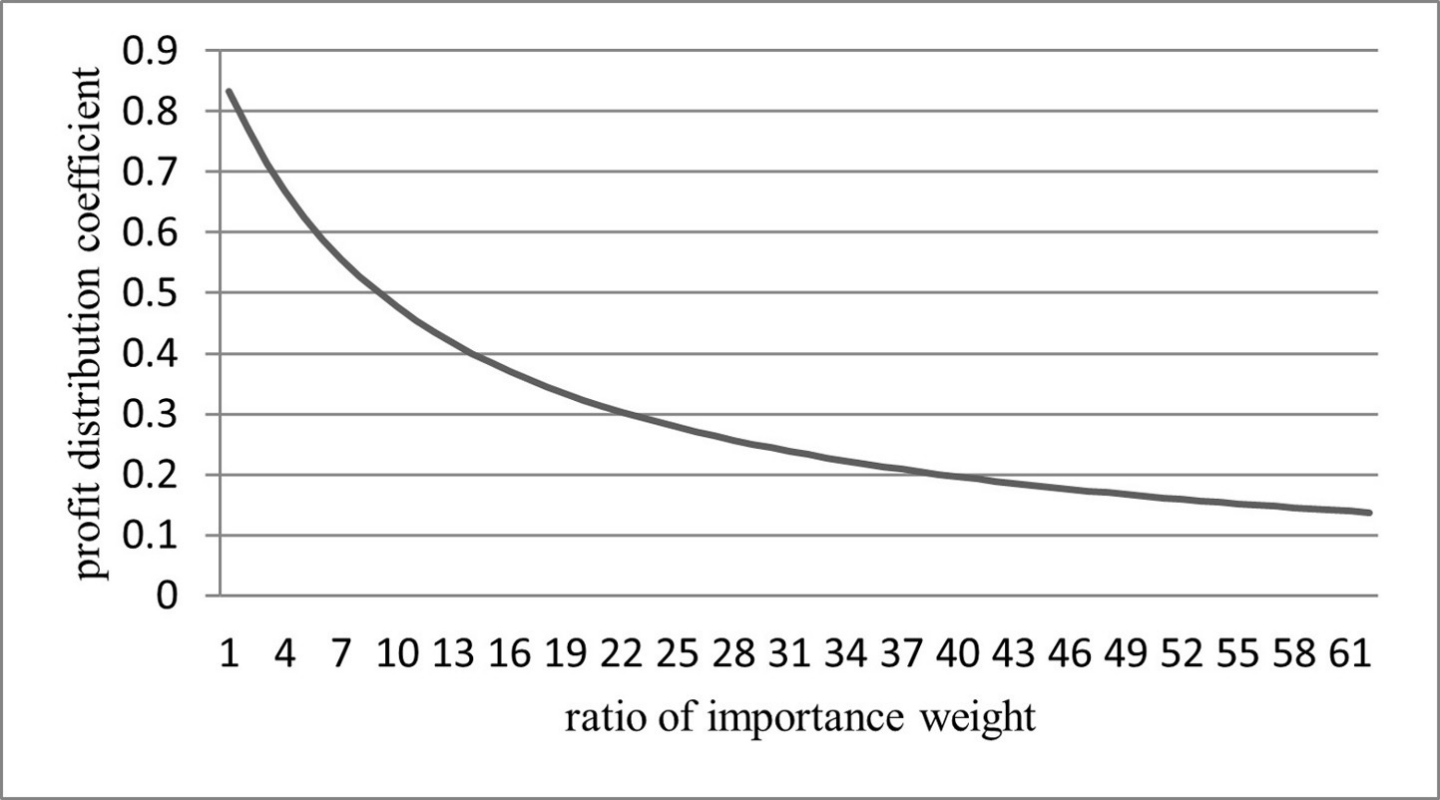
The curve of the profit distribution coefficient changing with the ratio of importance weight.

If $\frac{C_{2}}{C_{1}}=k=1$, the functional relationship curve between *φ* and $\frac{\alpha_{2}}{\alpha_{1}}$ can be obtained as shown in Fig 1.

**Proposition 2.** The ratio of profit distribution coefficient to input cost changes in the opposite direction, and the rate of change decreases.
**Proof.** Assume that the ratio of importance weight remains the same, i.e., $\frac{\alpha_{2}}{\alpha_{1}}=k$. It can be found that, as $\frac{C_{2}}{C_{1}}$ approaches zero, the profit distribution coefficient *φ* approaches one. It means that the cost of cooperatives is far greater than that of wholesalers in forming an integrated agricultural supply chain. Hence, cooperatives will get almost all of the excess profits.

On the contrary, as $\frac{C_{2}}{C_{1}}$ approaches infinity, the profit distribution coefficient *φ* approaches zero. It means that the cost of wholesalers is far greater than that of cooperatives in the integrated agricultural supply chain. Hence, wholesalers will get almost all of the excess profits.

**Fig 2:**
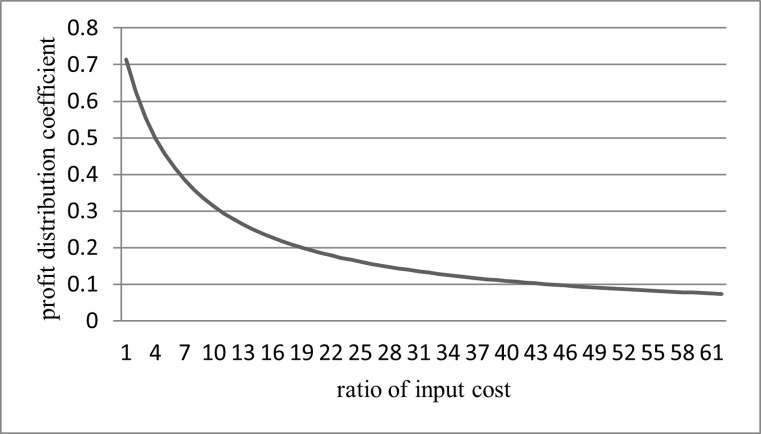
The curve of the profit distribution coefficient changing with the ratio of input cost.

If $\frac{\alpha_{2}}{\alpha_{1}}=k=2$ $\frac{{\text{C}}_{\text{w}}}{{\text{C}}_{\text{f}}}=0.5$, the functional relationship curve between *φ* and $\frac{C_{2}}{C_{1}}$ can be obtained as shown in Fig 2.

In order to verify the conclusion, the field research data of Qingfeng Green Onion Planting Professional Cooperative in Yulin Town, Lanxi County, Heilongjiang Province, which is a representative national cooperative in Heilongjiang Province, is used to conduct an example analysis. The cooperative has 58 peasant households, with a capital of about 5.7 million, mainly planting Chinese green onions, with an annual planting area of more than 7,000 mu, i.e., about 4.67 square kilometers. According to data from the cooperative, in the market demand *Q*(*P*) = *m* − *λP*, let *m* = 1200 kilogram, *λ* = 8, *C*_1_ = 0.4 yuan per kilogram, *P*_1_ = 0.9 yuan per kilogram, *C*_2_ = 0.2 yuan per kilogram, *P*_2_ = 5 yuan per kilogram. The calculation results are shown in Table 2.

**Table 2:** Example data analysis result.

*α*_1_	*α*_2_	*φ*	$\bar{{R^{\prime }}_{1}}$	$\bar{{R^{\prime }}_{2}}$
0.3	0.7	0.46	27471.21	17169.51
0.4	0.6	0.57	28697.61	15943.11
0.5	0.5	0.67	29760.48	14880.24
0.6	0.4	0.75	30690.5	13950.23
0.7	0.3	0.82	31511.1	13129.63

Note: The calculation result has two decimal places (unit: yuan)

As shown in the table above, as the importance weight of cooperatives in the integrated agricultural product supply chain increases, their distribution ratio of excess profits and the distributed profits are also increasing. The profit distribution model based on fair entropy can match the members’ benefits with their contributions, thereby improving their enthusiasm for participating in the supply chain formation, collaboration and operation.

## 6. Conclusion and suggestion

The agricultural supply chain is significantly different from the manufacturing supply chain in many aspects, which should be considered in the constructing of the profit distribution mechanism of the agricultural supply chain.

In this paper, aiming at the secondary agricultural product supply chain constructed by single cooperatives and single wholesalers, a profit distribution model under collective decision-making is constructed. Two factors of resources and risks are introduced to calculate the importance weight, and the profit distribution coefficient is calculated based on fair entropy and importance weight. We obtain the optimal solution, which means the fairest profit distribution model, and finally verify the model with an example. In summary, this paper draws the following conclusions:

1. Centralized decision-making through an integrated agricultural products supply chain is more capable of responding to market changes quickly and improving the competitiveness of the agricultural industry than decentralized decision-making. Whether the distribution of excess profits is reasonable is the decisive factor for each actor to form an integrated agricultural supply chain for centralized decision-making. The distribution of excess profits according to the optimal profit distribution coefficient can maximize the profits of each actor, which not only exceeds the profits under decentralized decision-making but also realizes the matching of returns and investment, that is, the most equitable distribution model.
2. This paper introduces fair entropy to solve the profit allocation coefficient. When the fair entropy reaches the maximum, the optimal solution is solved, and the excess profit is distributed fairly. The solution of the profit distribution coefficient is related to both the weights and input costs.

Based on the above research and conclusions, the following suggestions are made for the development of integrated agricultural supply chains:

1. China is a largely agricultural country but not an advanced agricultural country, which lies in the lack of high quality and efficient agricultural supply chain. A small-scale peasant economy has almost no advantage in the fierce market competition. We should consider the fair distribution of excess profits in the supply chain to make each actor voluntarily build a stable and long-lasting integrated supply chain model, centralize decision-making and participate in market competition. It is also the key to maintaining mutually beneficial and win-win cooperation.
2. Through contracts, property rights definitions and other forms, a precise distribution mechanism of profits and financial resources should be established so that each node unit can maintain high enthusiasm, and actively promote the development of the agricultural product supply chain. Participants in each the agricultural product supply chain link have different views and requirements. By establishing a scientific and reasonable benefit distribution mechanism, all stakeholders can negotiate in the same mechanism platform.
3. The profit distribution mechanism should take the common interests among participants as the premise. The best solution for distributing of benefits is negotiated among the participants and reinforced by agreements and contracts. In addition, the agreement cannot be separated from the legal constraints. Otherwise, the agreements and contracts will not be legally valid. When a conflict of interest occurs, more disagreement between the parties will occur. Thus, to protect the interests of all parties, the formulation of the contract should include both the form of benefit distribution and the risk prevention mechanism.
4. Profit distribution is not an even distribution, but to achieve a match between investment and return. In formulating the profit distribution mechanism and distribution coefficient, it is necessary to comprehensively consider the cost and importance weight of each actor’s input. For example, in the agricultural-supermarket docking, since the cost of supermarket input far exceeds that of the cooperative, the supermarket also occupies a dominant position in the supply chain. The excess profit gained by the supermarket should exceed that of the cooperative. Otherwise, the supermarket will withdraw or even destroy this integration decision-making model.
  Accordingly, to maintain the rights and interests of farmers, from the perspective of the market economy, it is necessary to increase the importance and voice of farmers in the supply chain. Farmers’ professional cooperatives are new organizations that adapt to the modernization, specialization and marketization of agriculture at the current stage. Through cooperatives, farmers can solve the problems of complex sales of agricultural products, difficult negotiations, and difficult access to capital and technology. Government departments should, on the one hand, widely publicize the successful experience of cooperatives, which can enhance the enthusiasm of farmers to join voluntarily. On the other hand, the government should regulate the development of farmers’ professional cooperatives and guide them to join the agricultural product supply chain operation, to protect the interests of farmers better. Therefore, effective coordination of subjects is the basis for fair profit distribution in the agricultural products supply chain.
5. It is also necessary to pay attention to information sharing, especially information on market demand. Without this information, farmers at the source of the supply chain will face huge losses. Government departments can develop incentives and subsidies to encourage large retailers to establish agricultural information systems. Government can also transmit information to farmers through television, newspapers, radio and other channels to reduce information asymmetry.

Compared with existing studies, this paper calculated an optimal profit distribution coefficient between the cooperative and the wholesaler, and uses the real data to verify and discuss the model. Some existing studies only compare the profit difference between the cooperation and non-cooperation [27], and some existing studies pay more attention on the influencing factors of profit distribution in the agricultural product supply chain [28]. Therefore, this paper conducts a further study based on existing research.

However, this paper still has some limitation, for example, the agricultural products supply chain only considers one cooperative and one wholesaler, and this paper only uses the data of one cooperative to conduct case study. In future, more stakeholders should be included in the supply chain, especially multi retailers, and more cooperatives should be used to verify the reliability of the model.

## Supporting information

S1 Dataset (DOCX)
