# Supplementary material for: Profit distribution mechanism of agricultural supply chain based on fair entropy
Source: PLoS One. 2022 Jul 25;17(7):e0271693. doi: 10.1371/journal.pone.0271693 (PMC9312423; doi:10.1371/journal.pone.0271693)
Supplement: S1 Dataset — (DOCX) [file pone.0271693.s001.docx]

**Table 2** Example data analysis result

| $\alpha_{1}$ | $\alpha_{2}$ |  | $\overline{R'_{1}}$ | $\overline{R'_{2}}$ |
| --- | --- | --- | --- | --- |
| 0.3 | 0.7 | 0.46 | 27471.21 | 17169.51 |
| 0.4 | 0.6 | 0.57 | 28697.61 | 15943.11 |
| 0.5 | 0.5 | 0.67 | 29760.48 | 14880.24 |
| 0.6 | 0.4 | 0.75 | 30690.5 | 13950.23 |
| 0.7 | 0.3 | 0.82 | 31511.1 | 13129.63 |

Note: The calculation result has two decimal places (unit: yuan)
